# The Design and Construction of an Electrohydrodynamic Cartesian Robot for the Preparation of Tissue Engineering Constructs

**DOI:** 10.1371/journal.pone.0112166

**Published:** 2014-11-18

**Authors:** Shaikh Hafeez Hashimdeen, Mark Miodownik, Mohan J. Edirisinghe

**Affiliations:** Department of Mechanical Engineering, University College London, London, United Kingdom; Imperial College London, United Kingdom

## Abstract

In this work we bring together replicating rapid prototyping technology with electrohydrodynamic phenomena to develop a device with the ability to build structures in three-dimensions while simultaneously affording the user a degree of designing versatility and ease that is not seen in conventional computer numerically controlled machines. An attempt at reproducing an actual human ear using polycaprolactone was pursued to validate the hardware. Five different polycaprolactone solution concentrations between 7–15 wt% were used and printing was performed at applied voltages that ranged from 1 to 6 kV and at flow rates from 5µl/min to 60µl/min. The corresponding geometrical and aesthetic features of the printed constructs were studied to determine the effectiveness of the device. The 15 wt% concentration at 60µl/min under an applied electric field of 6 kV was identified as the best operating parameters to work with.

## Introduction

A surge in demand for high throughput in the design and manufacture of complicated parts in a highly precise manner has paved the way for the development of advanced machinery that facilitates precision, control and consistency in production of these components. Computer Numerically Controlled (CNC) machines employ the use of advanced electromechanical systems that work synergistically to achieve this purpose. The hardware required to make this technology a reality has been gradually developing over the course of the last 30 years since the advent of the personal computer.

In recent times 3D additive manufacturing has appeared on the global stage as a major disruptive technology. The technology embodies itself in many competing ways, namely SLS (selective laser sintering) [1], FDM (fused deposition modelling) [1], [2], 3DP (three-dimensional printing) [1]–[3], LOM (laminated object manufacturing) and finally, stereolithography apparatus (SLA) [2]. Despite these processes having the ability to produce 3D structures, they lack consistency and accurate reproducibility. Techniques like LOM and SLA require additional preparation steps before the product is finished. These additional steps increase the complexity and the cost of fabrication.

The ease of use and cost had always been major drawbacks that have in the past isolated 3D printing technology only to a niche market. However, reduction in cost of hardware and innovative programming has led to accessible open source projects like RepRap which has marked a peak in the steady maturity of this technology over the last three decades [4].

At present Reprap which is an FDM technology has been portrayed only as a novelty device with extensive and versatile printing capabilities at very coarse resolutions. Unfortunately, in-situ material manipulation for printing is only limited to thermoplastics that can be heated just beyond their glass transition temperature before being extruded onto a heated bed. Metals and heat sensitive organic compounds are yet to be printed via FDM (fused deposition modelling) [5]. Moreover printing onto surfaces is restricted to the heated bed that is supplied with the printer. Nevertheless, attempts have been made to use the technology to build specialised 3D printers that can be used to extrude cell solutions and hydrogels accurately to manufacture scaffolds for organ regeneration with varying degrees of success. The technology allows for spatially heterogeneous multi-material structures to be produced resulting in cell laden scaffolds for applications in replacement knee menisci and invertebral discs [6]–[8].

Congenital microtia or auricular traumatic amputation remains till today as a major challenge to plastic surgeons [9]. Work done by Cao et al. [10] described a successful generation of cartilage in the shape of a human ear in a nude mouse. This was achieved by impregnating a biodegradable polyglycolic acid mesh with bovine cartilaginous cells and then placing the mesh on the back of a mouse. Gradual improvements over the years in tissue engineering and 3D reconstruction technologies has led to encouraging attempts at in vitro engineering of constructs capable of maintaining the intricate architecture of the auricle while being anatomically refined. This was exhibited by work done by Reiffel et al. [11] who repeated work attempted by Cao et al. [10] in 1997 but instead of using a mould they designed their auricle scaffold using a 3D printer. Studies conducted by Mannoor et al [12] took it a step further by printing a bionic ear in an effort to merge biological tissues and functional electronics to create a cyborg organ that is capable of improved auditory sensing.

PCL (Polycaprolactone) is a semi-crystalline, bioerodable polymer that possess the ideal mechanical properties that are necessary for it to be employed for use as a temporary extracelluar matrix for cartilage regeneration. It also possess an ideal rate of degradability in favour of new ECM growth when implanted or cultured in a bioreactor. It dissolves in most organic solvents and is capable of undergoing hydrolysis within the body to form water soluble monomers. As a result the polymer has found its way into a number of biomedical devices for applications that include implantable contraceptive devices and staples for wound closure that degrade over time [13], [14].

Electrohydrodynamic direct writing is a flexible cost effective alternative technique that is capable of producing a very fine stable jet of liquid in the presence of an external electric field [15], [16]. This jet can then be used to pattern surfaces in an ordered and controlled fashion and offers a robust route to low cost large area micro and nano-manufacturing. Unlike other types of direct writing techniques, the liquid in electrohydrodynamic printing is subjected to both pushing and pulling forces. The pushing force is due to the constant flow rate that is maintained via high precision mechanical pumps while the pulling force occurs due to a potential difference that is applied between the nozzle and the ground electrode and as a result a fine jet can be generated to pattern surfaces. [17].

In stark contrast to fused deposition modeling, the electrohydrodynamic technique is not hindered by the material and is capable of depositing a variety of materials at any viscosity without the risk of clogging. Metals (copper, silver, gold), ceramics and inorganic/polymers can be processed into solution and printed at very high resolutions in both the micro and nano-scales [18]–[21]. In addition, EHD printing can also take place on a variety of surfaces provided the surface is grounded. At present, creating intricate and irregular shaped 3D structures have not been attempted with this technique.

Thus this article describes the design and construction of a new electrohydrodynamic printing machine with the sole purpose of expediting research in electrohydrodynamic printing in a flexible, feasible and user friendly manner. To achieve this, rapid replication technology is merged with conventional electrohydrodynamic printing phenomena to produce a high resolution EHD printing unit. In order to validate our work the work also describes an attempt to print a fully formed human ear out of polycaprolactone.

## Materials and Methods

### Solution preparation

PCL (molecular weight 80 kDa) and dimethyl carbonate were both purchased from Sigma-Aldrich. PCL pellets were dissolved in DMC to makeup solutions with five different concentrations of polymer 7%, 10%, 12%, 14% and 15% (by weight). This was done to identify the best concentration for dispensing and ease of solvent evaporation that would result in a solid structure. PCL pellets and DMC were placed in a glass bottle along with a magnetic stirrer and the solutions were magnetically stirred at ambient temperature of 25°C for three hours, which was needed for complete dissolution.

### Characterisation of PCL solutions

Key solution characteristics such as (electrical conductivity, surface tension, viscosity and density) were measured since they are critical for steady electrohydrodynamic jetting to take place. All of the equipment used to take measurements were initially calibrated and subsequently measurements of the PCL solutions were taken (Table 1) at the ambient temperature (25°C), pressure (101.2 kPa) and humidity of (50–55%). Electrical conductivity readings were taken using a Jenway 3540 pH/Conductivity meter (Bibby Scientific Limited, Stafford, UK) while the surface tension was measured using a Kruss tensiometer (Standard Wilhelmy's plate method). The viscosity was measured using a Brookfield DV-III Ultra Rheometer for small volumes with a SC4-18 spindle (Brookfield Viscometers Ltd, Harlow, UK).

**Table 1:** Physical properties of PCL solutions used in the experiments.

Solution	Density(kgm^−3^)	Viscosity (mPa s)	Electrical conductivity (S m^−1^)	Surface tension(mNm^−1^)
Dimethyl Carbonate	1.08	1.44±0.2	4.3×10^−9^	30.5±0.3
PCL 7%	1.10	26±0.2	4.3×10^−9^	31.2±0.6
PCL 10%	1.13	34.3±0.2	4.9×10^−9^	32.5±0.2
PCL 12%	1.16	73.7±0.1	5.2×10^−9^	33.3±0.8
PCL 14%	1.18	133.5±0.2	5.5×10^−9^	35.3±0.7
PCL 15%	1.20	192.8±0.4	6.0×10^−9^	40.3±0.5

All % refer to weight.

### The Setup

**Figure 1:**
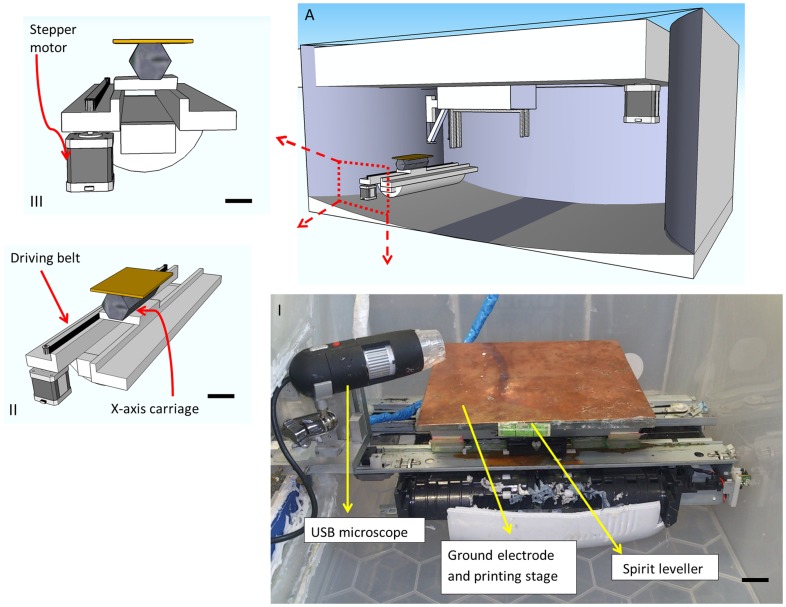
Front view of the printer (x-axis). A) internal view of the printer A(I)The X-axis and the components with respect to each other A(II) isometric view of the x-axis showing the drive belt and x-axis carriage A(III) side-on view of the x-axis showing how the stepper motor fits into the x-axis. (Scale bar  = 100 mm)

**Figure 2:**
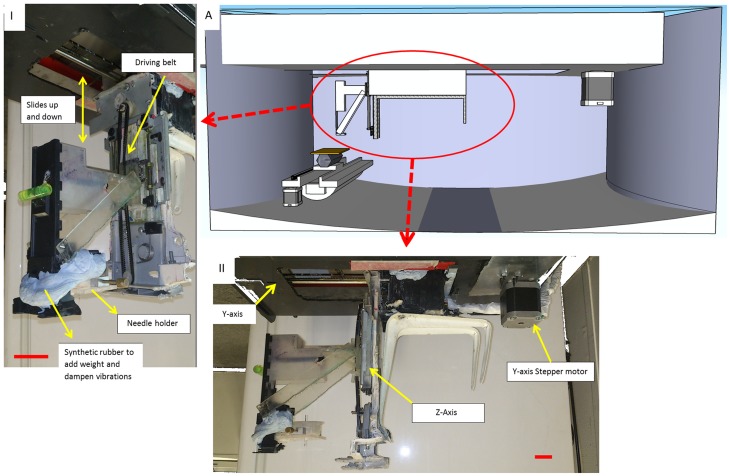
Cross-sectional view of the printer revealing the Y and Z axis assembly within the printer. A(1) key components of Z- axis, gears, driving belt and needle holder A(II) side on view showing the positions of Y and Z axis in relation to each other. (Scale bar  = 20 mm)

**Figure 3:**
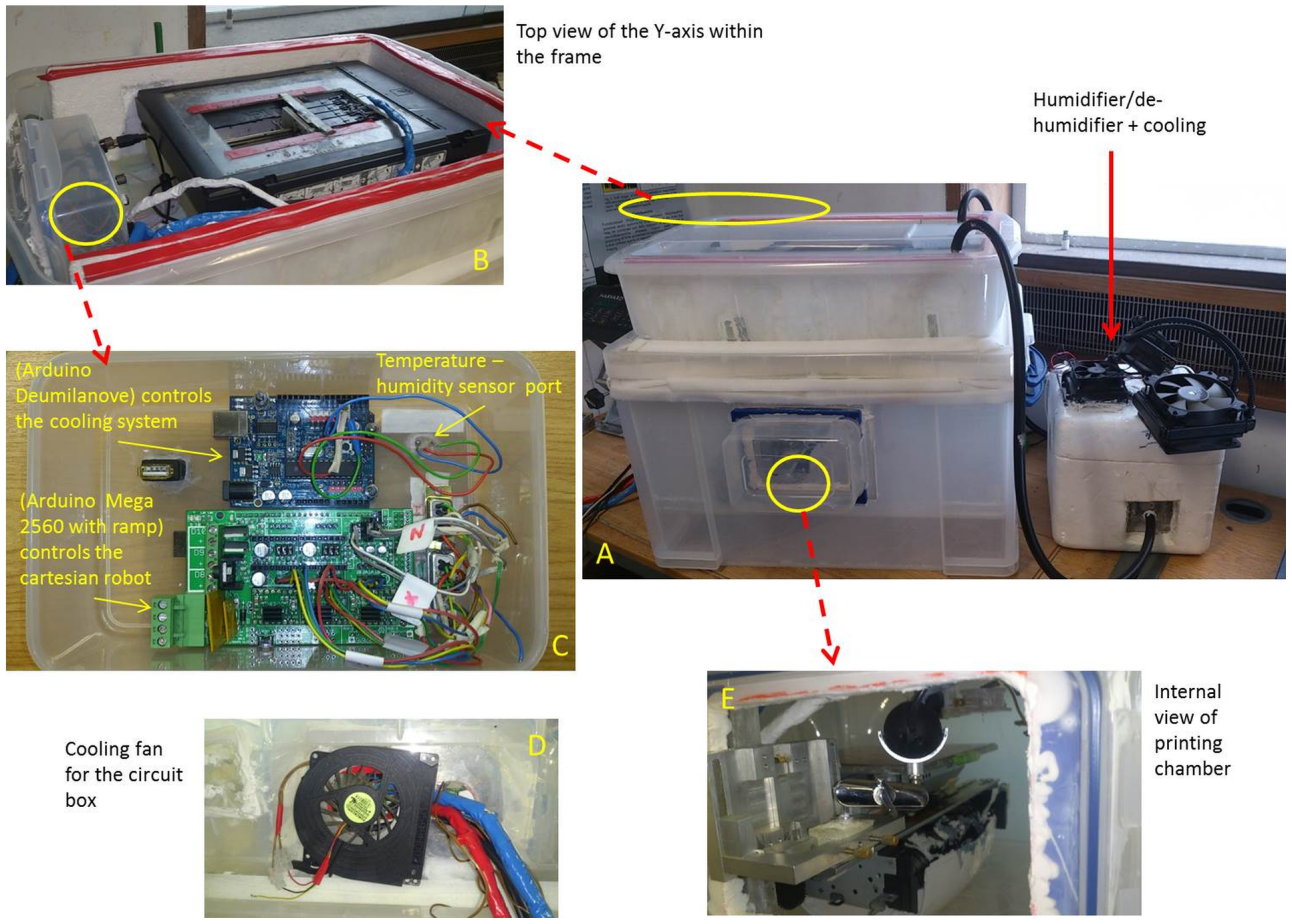
The Cartesian robot. A) full setup B) y-axis cradle C) main circuit box D) cooling fan for the main circuit box E) Internal view, x and y axis positions can be seen in relation to each other.

The EHD 3D printer that was designed and constructed in our laboratory is a purpose-built cartesian robot, using the popular open-source Arduino microcontroller as its central feature together with stepper drivers, motors, and sensors to control its movements (Figure 1–3). To perform a print, a CAD (computer aided design) file that contains the model design is first converted into an STL (standard tessellation language) file. The STL files contain three-dimensional polygons that are sliced up into distinct layers using a slicing software so that the printer can easily digest its information. The program then divides the object into digital cross-sections so that the printer is able to build structures layer-by-layer. The cross-sections essentially act as guides for the printer, so that the object is the exact size and shape, achieved by specifying tool paths to dictate precise movements. This is then followed by conversion into machine understandable G-code that can be used to control the stepper motors which in turn determine accurate translations in the x, y or z direction.

A script based software determines the tool path from the STL file and sends the information to the Arduino 2560 mega microcontroller which in turn interfaces with the reprap arduino mega pololu shield to drive the stepper motors which were preferred over servo motors due to the low operating accelerations and static loads that each axis was expected to endure during the course of each print. The stepper motors fit into an open loop control system where no real time feedback is provided with respect to the home positions of each axis. This apparent drawback is offset by a new feature called microstepping that facilitates each step taken by the motor to be subdivided further into smaller micro steps thus enabling high precision and accuracy during motion. This microstepping feature which is provided by the A4988 pololu stepper drivers also serves to allow a stepping motor to stop and hold a position between the full or half-step positions. This feature also eliminates the jerky character of low speed stepping motor operation thus reducing vibrations and diminishing problems related to resonance.

When operating at low speeds there is always a risk of the stepper motor missing steps due to a fall in current followed by a sudden subsequent rise of current in the copper windings of the motor. An enhancement designed into the A4988 pololu stepper driver specifically addresses this issue by mixed decay operation which prevents rapid fluctuations in current to ensure smooth motion regardless of changes in speed during a print.

### Machine frame and axes

A sturdy polypropylene structure was used to bear the static and dynamic loads, thermal strains and mechanical vibrations. It is essential that the polypropylene structure does not deform or vibrate under the action of forces encountered in printing. All components of the machine must remain in correct relative positions to maintain the geometric accuracy while printing, regardless of the magnitude and direction of the forces encountered during operation. Two polypropylene boxes were used to build the frame of the electrohydrodynamic cartesian robot. A closed chamber was preferred to ensure maximum isolation from the external environment.

**Figure 4:**
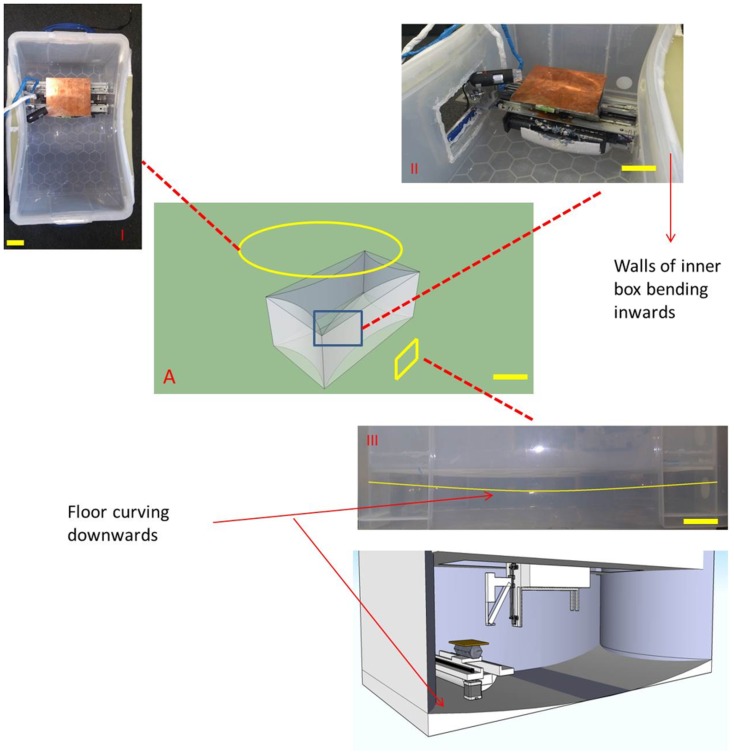
Polypropylene frame. 1) top view showing contours of the internal box II) inward bending of wall III) floor bending downwards. (Scale bar  =  300 mm)

The walls of the inner polypropylene box bend inwards to reduce the area of contact with the external box (Figure 4). This is done to minimise flow of heat and vibrational energy into and out of the printing environment. The configuration also forces the inner box into a state of compression thus making the frame sturdy with the external box forming the tough flexible exo-skeleton. Within this frame, three belt driven axes are placed orthogonally to each other with the Z axis hanging off the X-axis (Figure 2). All three axes were fitted with mechanical endstops to feedback information to the computer on the limits of each axis.

During printing the Z-axis makes 100µm translations after every layer is deposited while the x-axis and y-axis are capable of delivering up to a minimum of 10µm translations at a time. This represents an overall increase in the printing resolution when compared to work that was performed on a conventional CNC machine [22] where the resolution was 24µm.

The printer also features a flat ground electrode made from copper (Figure 1). This enables the electric fields lines generated by the positively biased needle to meet the ground electrode perpendicularly. This is done to improve homogeneity of the electric field lines close to the surface which should serve to minimise whipping and axisymmetric instabilities in the jet during printing. A generic USB microscope is installed to provide live video feed during any print. The USB microscope is capable of recording in high definition at 30 frames per second.

### Fluid deposition

A Harvard syringe perfusor pump was used to deliver the PCL solutions to the print head through silicone tubing measuring 300µm in diameter. The needles were connected and regulated via a high voltage power supply (Glassman Europe Ltd, Tadley, UK). The structures were printed into petri dishes filled with distilled water. To avoid charge build up, the petri dishes were grounded using an external ground electrode.

### Cooling/humidity control system

Since temperature and humidity play a vital role in achieving a stable cone-jet, it is necessary for the operating environment to remain at a specified temperature and humidity depending on the type of polymer that is used in the print. An automated cooling system was built to maintain a constant temperature and humidity for the duration of the print which usually lasts for about 190 minutes.

**Figure 5:**
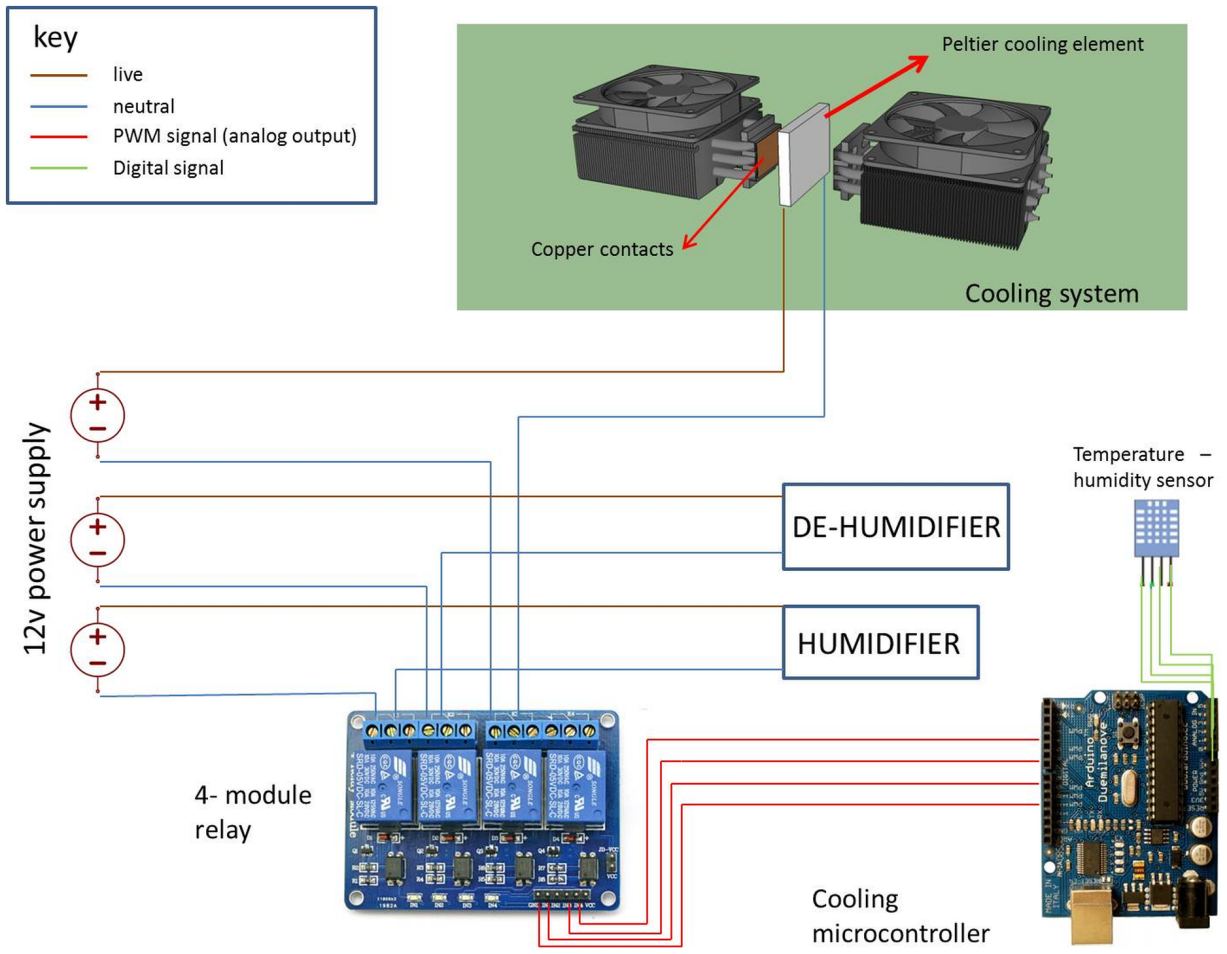
Circuit diagram illustrating the connections between the microcontroller, humidifier, de-humidifier and the cooling mechanism.

The mechanics of automated cooling control requires a simple feedback loop to be in place to ensure efficient regulation. In this feedback loop, a DHT11 temperature-humidity sensor is placed close to the printing area. The sensor reads the current temperature and humidity of the chamber and feeds the information back into a program written in C language which is installed and running on the microcontroller. The readings are then matched to a predefined setting which is specified by the user and subsequent humidity levels and cooling are regulated via the engagement of the peltier cooling element, humidifier/de-humidifier as and when required. This is made possible through a series of connections between the respective devices and the power supply through a relay module that is in turn controlled by the microcontroller (Figure 5). The relay module acts as switch, switching on and switching off any of the devices wired up to it based on instruction from the microcontroller.

**Figure 6:**
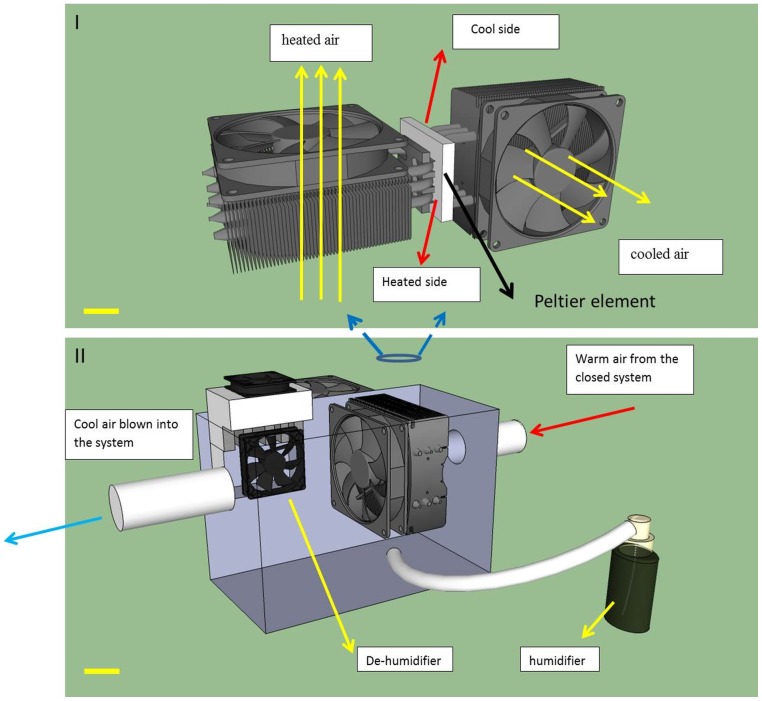
The cooling/humidity control system. I) how the cooling mechanism works II) cross-sectional view illustrates the different components of the cooling/humidity control system. (Scale bar  = 30 mm)

The cooling system consists of a peltier cooling element sandwiched in between two water liquid coolers (Figure 6 (I)). The peltier element uses the peltier effect to generate a heat flux at the juncture between the two sides of the element which is made of two different types of materials. This peltier effect results in a temperature differential of about 60°C between the two surfaces of the element (Figure 6(I)). As a direct result of this the peltier element can act as a solid state thermoelectric heat pump that is capable of moving heat energy out of the printing chamber. The cool side of the peltier element is placed in contact with the copper contacts of the water cooler across which water (the coolant) is pumped before being channelled back to the radiator where the air from the printing chamber is cooled by making it to pass across chilled aluminum fins before being directed back into the main chamber (Figure 6 (I) and (II)).

## Results and Discussion

**Figure 7:**
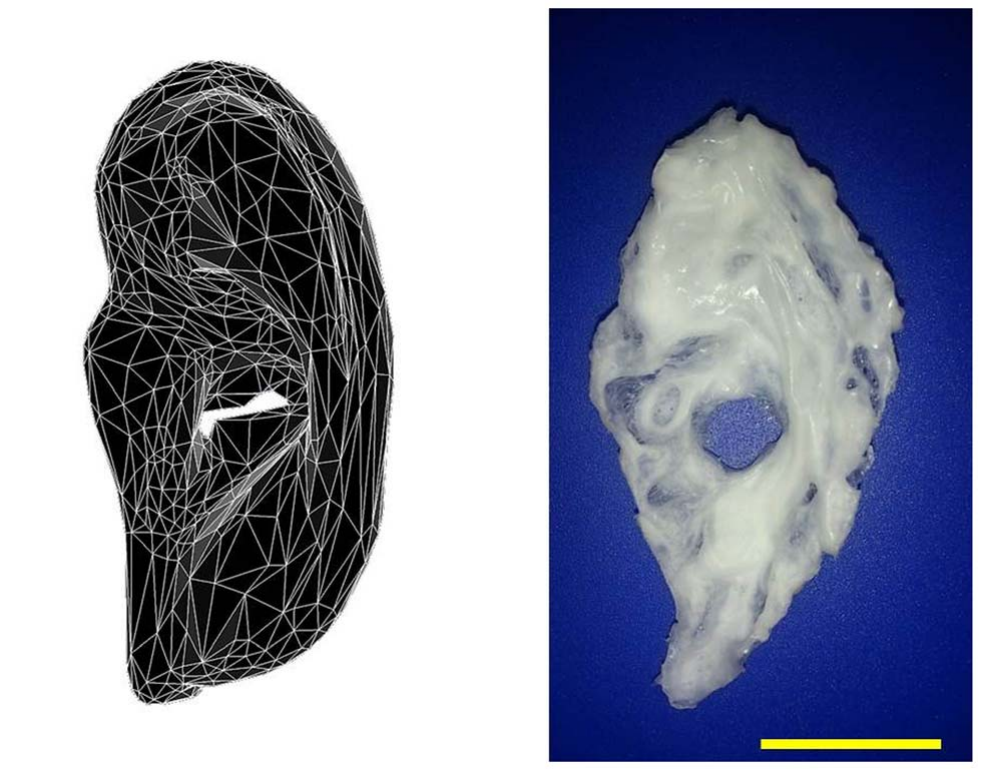
The three-dimensional STL construct compared to the actual printed construct (scale bar  = 15 mm).

The design and construction of new hardware warrants an investigation to demonstrate its capabilities and areas of improvement. Thus the system validation for this machine involves the fabrication of a human ear (Figure 7). Thin continuous micrometer sized jets are pulled out of a meniscus suspended from a print head with a 800µm nozzle in the presence of an high electric field. This fine jet represents a large ratio of jet diameter to nozzle diameter and is used to fabricate the 3D construct in this study. The printing takes place within a petri dish filled with distilled water that offers a lucid cushion like surface to print on. As layer after layer of PCL is deposited, an equal volume of water in the petri dish is displaced thus allowing the construct to grow with minimum resistance. The petri dish was grounded to ensure that the positive charges being delivered to the petri dish by the electrohydrodynamic jet during the printing process were neutralised.

**Figure 8:**
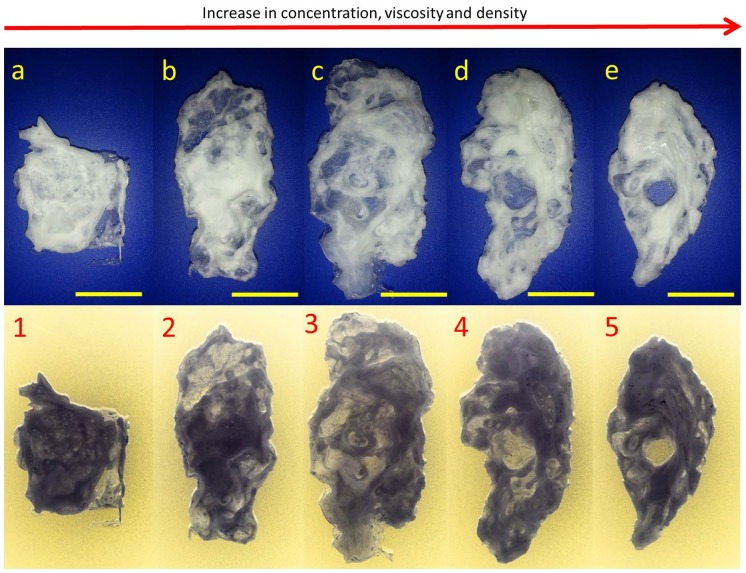
The gradual evolution of the construct with respect to concentration, viscosity and density (scale bar 20 mm).

For the five PCL solutions that were investigated, we can see from (Figure 8 a–e) the gradual evolution of the intended construct from a structure that possess no resemblance to a human ear (Figure 8a) to a stable 3D human ear construct (Figure 8e). The use of a grounded water bath ensured the rapid precipitation of the PCL which is mainly due to PCL's high hydrophobicity for water. This precipitation became more prominent with the increase in PCL content in the printing solution. Printing solutions with lower concentrations (7%, 10%) tended to spread along the surface of the water bath as they were deposited which is illustrated in (Figure 8, 1–3) where the dark, condensed portions indicate where the material has been deposited in significant amounts. The increased solvent to solute ratio made it harder for the precipitated PCL to remain in place because as the solvent DMC was taken up in water it would carry the PCL away with it. Thus shape integrity and uniformity was difficult to control when using low concentrations of PCL.

At high concentrations (15%,14%), the first few layers deposited were more than enough to anchor down the construct and offer a firm printing surface onto which subsequent layers of PCL could be deposited. The higher viscosity also contributed towards distinct features like the inner folds of the ear to become more pronounced (Figure 8e and 8(5)).

**Figure 9:**
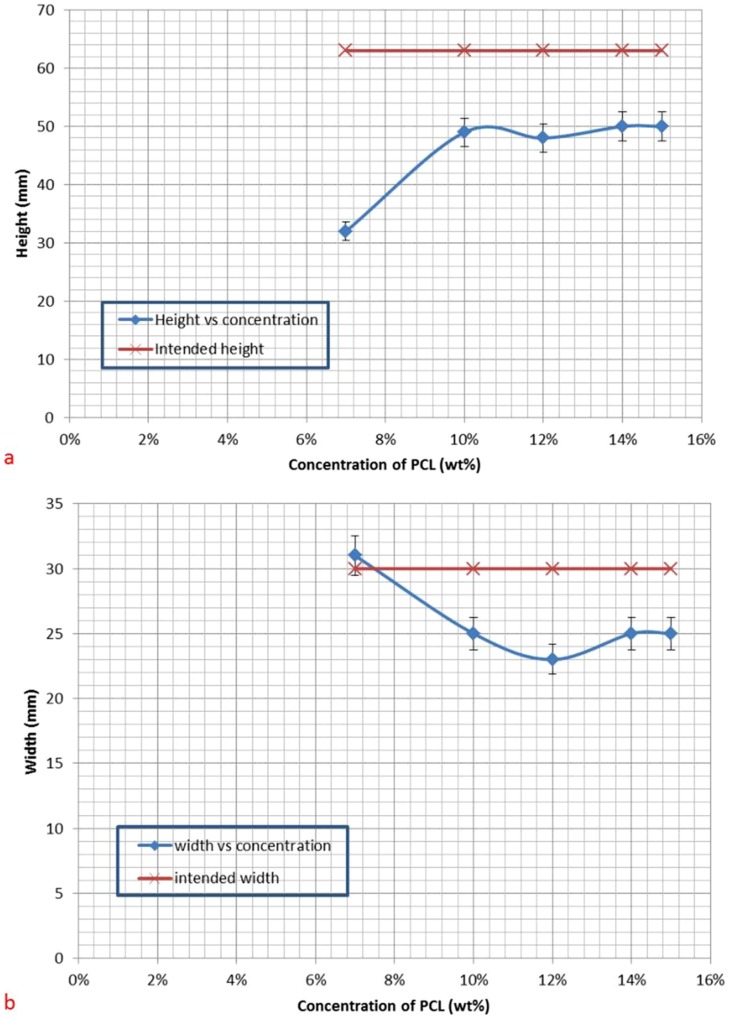
A comparison of the shrinking observed in the constructs. a) width b) height.

**Figure 10:**
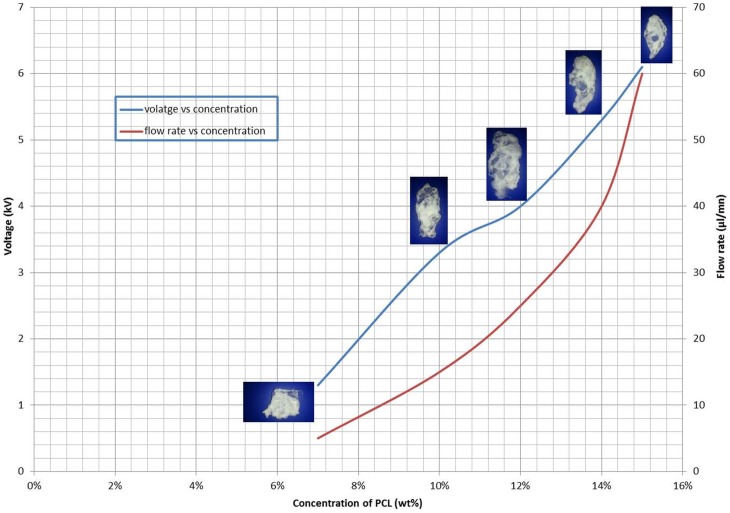
The operating envelope for printing.

Construct shrinking was an issue with all prints. Almost all the constructs contracted in height (20–23% reduction in height) and width (11–16% reduction in width) as the solvent evaporated, see fig (9(a, b)). The shrinking was more severe in the vertical plane with a significant reduction in thickness. This is a direct result of the low PCL content that was used in the study. Concentrations exceeding 15% proved to be too viscous to work with since they often solidified within the silicone tubing and clogged up the print head during the printing.

(Figure 10) illustrates the ideal operating regimes within which a stable 3D construct can be printed while maintaining a continuous stable jet in the cone-jet mode for the duration of the print (190 minutes). The higher viscosity solutions (15%, 14%, and 12%) required a higher electrostatic force to overcome the surface tension at the meniscus for jetting to take place. The increased PCL content would have restricted the mobility of charged ions and molecules and therefore to overcome this, higher voltages (4 and 6 kV) and higher flow rates (40 and 60µl/mn) were required for 14% and 15% solutions. The high flow rates and low standoff heights of (30 mm) served to force the continuous jet along a linear path to the surface of the water bath, thus preventing the chances of varicose and whipping instabilities from arising in the jet.

**Figure 11:**
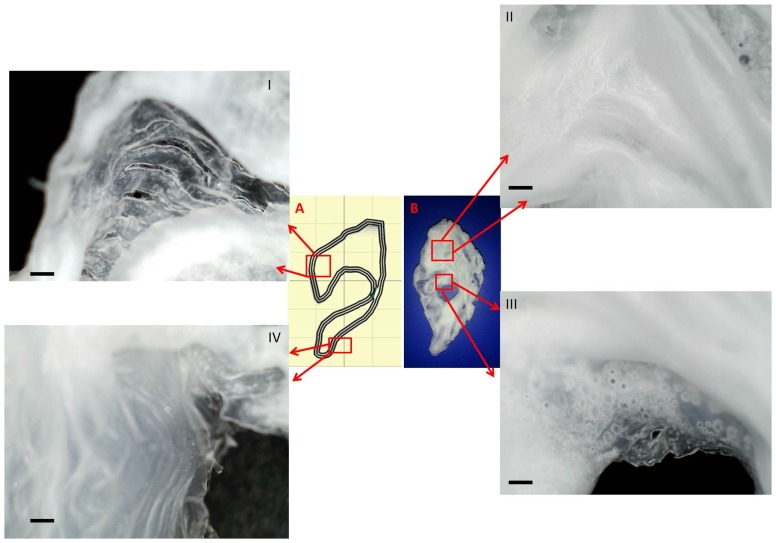
Close up examination revealing key features of the construct. a) live screen capture of the tool path taken during the print b) actual construct. (Scale bar  = 1 mm).

Close up examinations of the printed constructs revealed that successive layer by layer deposition was accurate as can be seen in (Figure 11-I). Similarly when printing with the high viscosity solutions (15% and 14%), minute air bubbles were found trapped at certain points in the constructs (Figure 11-IV). This only occurred when the print was focused on an area or region that was intended to be thicker or required more material for extra support.

## Conclusions

This paper highlights the development of an electrohydrodynamic printer with the capability of controlled deposition at a range of flow rates, applied voltage and printing solution viscosities. The union of RepRap technology with Electrohydrodynamic phenomena has proven to offer greater design freedom and geometrical versatility for EHD printed constructs when compared to attempts reported in literature. The study has also highlighted the need for the optimization of the material formulations for accurate and instantaneous deposition of material during the printing process. The system would benefit from a real time feed-back loop to compensate for the effect of intrinsic material property limitations on maintaining a consistent jetting for lengthy printing durations. This would also help bridge the gap between the actual printed constructs and the idealised three dimensional objects that are drawn up.
